# Solitary anterior abdominal wall leiomyoma in a 31-year-old multipara woman: a case report

**DOI:** 10.1186/1757-1626-2-113

**Published:** 2009-02-01

**Authors:** Gabriel O Igberase, Tagbire O Mabiaku, Peter N Ebeigbe, Harrison O Abedi

**Affiliations:** 1 Department of Obstetrics and Gynecology, College of Health Sciences, Delta State University, Abraka, Delta state, Nigeria; 2 Department of Family Medicine, College of Health Sciences, Delta State University, Abraka, Delta State, Nigeria

## Abstract

**Background:**

Anterior abdominal wall fibroid are uncommon and could be a cause of pain and discomfort. Very few cases have been reported in the literature but none in our region.

**Case presentation:**

We present an uncommon case of a 31 year old para 2+2 trader from the Itsekiri tribe of the Niger Delta region of Nigeria who presented with a one year history of a periumbilcal mass, had surgical removal of the mass and histology revealed leiomyoma.

**Conclusion:**

Abdominal wall fibroid is a good differential diagnosis to be considered in any woman of reproductive age with an anterior abdominal mass and previous uterine surgery, including laparoscopic surgeries.

## Background

Fibroids also called leiomyoma are said to be the commonest benign tumour of the reproductive tract and are clinically apparent in 20% of women of reproductive age. The may be present in as many as 70% of uteri removed at hysterectomy [1]. The commonest site is the uterus but they are also found in the broad ligament, ovaries, vagina and rarely on the anterior abdominal wall [2-5]. Uterine fibroids are associated with infertility, menorrhagia, pain and compression symptoms when very large [2,6]. Abdominal wall fibroids are an uncommon finding and are thought to follow seeding following surgical resection of uterine fibroids [4,7,8]. With the advent of laparoscopic myomectomies, more cases of abdominal wall fibroids are now being reported.

We present an unusual case of abdominal wall leiomyoma in a 31 year old multipara with 2 previous Caesarean sections and no evidence of uterine fibroid.

## Case presentation

We present a case of a 31 year old para 2+2 (3 alive) female trader from the Itsekiri tribe in the Niger Delta region of Nigeria. She was first seen at the gynaecological clinic with a one year history of progressive periumbilical swelling which later became associated with pain. The mass at initial presentation measured about 2.0/1.5 cm and a year later became 10.0/8.0 cm. It was located on the left periumbilical area, roundish, mildly tender, firm, slightly mobile and not attached to the skin or underlying tissue. She has 3 living children having had a set of twin and both deliveries were by Caesarean section. Indications for her 2 previous Caesarean sections were twin gestation and cephalopelvic disproportion respectively There were no findings of uterine fibroids from previous ultrasound scans and Caesarean sections done. Abdominopelvic scan done at presentation was essentially normal except for the anterior abdominal wall mass measuring 100 mm/96 mm of mixed echogenicity suggestive of anterior abdominal wall cyst or fibroid.

**Figure 1:**
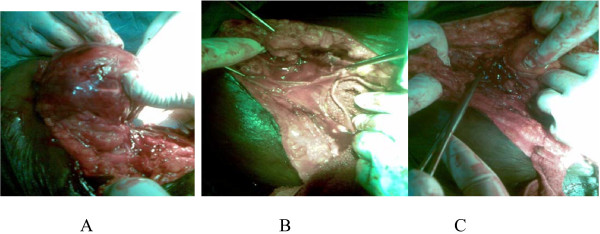
**1a, showing abdominal leiomyoma being disessected from the subcutaneous tissue, 1b shows the fibroid bed and 1c shows a bowel loop being pushed in after inspection of peritoneum**.

Her general clinical state was normal. She was counseled for surgery. The abdomen was entered via a previous midline scar which was extended slightly above the umbilicus to reach the mass. The incision was developed into the subcutaneous tissue and rectus sheath and the mass was enucleated from its capsule (figure 1), the pelvic organs were inspected and found to be normal. Findings at surgery were a periumbilical mass situated between the subcutaneous tissues and the rectus sheath, its capsule also attached to the rectus abdomnis muscle, mild pelvic adhesions, normal uterus with no fibroid mass, apparently normal fallopian tubes and ovaries. Estimated blood loss was 400 mls.. The fibroid bed was repaired and abdomen closed in layers with interrupted nylon to skin. Histology of the mass revealed leiomyoma.

## Discussion

There is paucity of findings of isolated abdominal wall fibroids in the literature without previous surgeries for myomectomies or presence of uterine fibroids. She has never been managed for infertlity nor has any diagnosis of uterine fibroids been made. Indications for her 2 previous Caesarean sections were twin gestation and cephalopelvic disproportion respectively. There was no evidence of uterine fibroids in all surgeries. This supports the thinking that leiomyomas can be found anywhere there are smooth muscles [1].

Abdominal wall fibroids are commoner after laparoscopic surgeries are done compared to open surgeries [4,7,8]. Incarceration of a sessile uterine fibroid in an umbilical hernia during pregnancy has been described [3].

This is the first reported case of abdominal wall leiomyoma in the medical literature from this region. Laparoscopic myomectomies are not widely done in our country because of lack of skills so many gynaecologist resorts to open myomectomies. This may be the reason why it is not common. The uniqueness of this case is the fact that the patient does not have uterine fibroid. There is the possibility that uterine seeding into the abdominal wall could have occurred in one of the Caesarean sections.

Surgical removal was the mode of treatment and she did well postoperatively with disappearance of symptoms of pain.

## Conclusion

In conclusion, abdominal wall fibroid is a good differential diagnosis to be considered in any woman of reproductive age with an anterior abdominal mass and previous uterine surgery, including laparoscopic surgeries.

## Consent

Written informed consent was obtained from the patient for publication of this case presentation and accompanying image. A copy of the written consent is available for review by the editor-in-chief of this journal.

## Competing interests

The authors declare that they have no competing interests.

## Authors' contributions

**GO **did the recent surgery and the second Caesarean section, provided data read and approved the manuscript. **TO **assisted recent surgery and the second Caesarean section, provided data read and approved the manuscript. **PN **provided data read and approved the manuscript. **HO **provided data read and approved the manuscript
